# Ex vivo culture of keratinocytes on papillary and reticular dermal layers remodels skin explants differently: towards improved wound care

**DOI:** 10.1007/s00403-019-01941-w

**Published:** 2019-06-05

**Authors:** Timothy Bage, Trevor Edymann, Anthony D. Metcalfe, Baljit Dheansa, Lubinda Mbundi

**Affiliations:** 1 Faculty of Medicine, University of Southampton, University Road, Southampton, SO17 1BD UK; 2 Blond McIndoe Research Foundation, Queen Victoria Hospital, Holtye Road, East Grinstead, RH19 3DZ UK; 3 Healthcare Technologies Institute, School of Chemical Engineering, Institute of Translational Medicine, University of Birmingham, Mindelsohn Way, Edgbaston, Birmingham, B15 2TH UK; 4 Plastic Surgery and Burns, Queen Victoria Hospital, East Grinstead, RH19 3DZ UK; 5 Department of Surgical Research, Northwick Park Institute for Medical Research, University College London (UCL), Northwick Park & St Marks Hospitals, Watford Road, Harrow, HA1 3UJ UK

**Keywords:** Papillary, Reticular, Keratinocytes, ECM

## Abstract

In this study, we characterised the effect that seeding keratinocytes on the papillary and reticular dermis had on the extracellular matrix and tissue integrity ex vivo. Human skin explants from consented patients (*n* = 6) undergoing routine surgery were cultured at a liquid–air interface, dermal-side up, and autologous keratinocytes seeded on the exposed papillary or reticular layer. After 7–21 days, histological and immunohistochemical evaluation of the morphology and extracellular matrix was performed. While the dermis remained robust in all explants cultures, keratinocytes seeded on the papillary layer showed less tissue infiltration and remodelling and formed clusters across the tissue. In contrast, keratinocytes seeded on the reticular layer infiltrated the tissue homogenously with an intact single-cell-layer surface coverage and structural changes characterised by increased deposition of ground substance, glycosaminoglycans, and collagen VII in 14 days. In addition, while the papillary section showed more new laminin deposition by 14 days than the reticular section, the latter expressed more connexin 43. These differences in re-epithelialisation and extracellular matrix characteristics suggest that wound depth and graft thickness may play a key role in wound healing and indicate that ECM characteristics should be factored in when designing biomaterials for wound applications and in the selection of recipient sites when using cells for grafting.

## Introduction

The interactions between dermal cells and the extracellular matrix (ECM) are vital in maintaining the integrity of skin and would healing [14]. Structurally, the ECM is composed of the fibrous proteins (i.e., collagens, elastin, and laminin) that give skin its robust tensile strength alongside elasticity, and the highly hydrated proteoglycans made of glycosaminoglycan (GAG) chains branching off linear protein cores [10, 14]. The variation in the ECM and cell-integrin expression across the regions of the skin distinguishes various environments that recruit and modulate the properties and activities of different cell types [14]. In this regard, the fibroblasts and the ECM which they produce has been shown to be characteristically different between the papillary and the reticular dermal layers [6].

In light of the diverse ECM profile, studies in wound healing and dermal substitute materials have focused at recapitulating normal wound healing and skin [3]. In this regard, the use of in vitro monolayer cultures of human skin cells to understand cellular mechanism involved in wound healing is limited by the absence of the multidimensional 3D environment physico-chemical cues [1]. While it is well accepted that in vivo studies in humans offer a conclusive testimonial of the cell-ECM crosstalk that is efficacious for clinical applications, such studies present several practical, ethical, and moral concerns. As such, in vivo studies in animal models have received increased attention as they allow for exhaustive analysis of the cellular and ECM interaction in living tissue. However, the clinical efficacy of using animal models, such as murine and porcine, which have different skin and healing profile to that of humans remains unclear [1]. As such, in vitro studies using organotypic and 3D human skin equivalents seeded with either keratinocytes or fibroblasts or both are widely used [5, 8] to bridge the gap between in vitro and in vivo methods. However, although these models provide a multidimensional environment around cells, the matrices are simplified with limited fidelity to human skin in vivo [5, 8].

On the other hand, the use of human full- and partial-thickness ex vivo culture allows the study of particular cell activities within an intact skin ECM. Indeed, human skin ex vivo culture systems have been employed in several dermatological and wound healing studies [5, 8]. In this study, to elucidate the effects of human epidermal keratinocytes on the ECM features in the dermis, skin explants were cultured (dermal-side up) and seeded with keratinocytes at the air–liquid interface, and histological and immunohistochemical staining for the cells and components of the ECM were performed.

## Materials and methods

### Cell isolation and culture

Healthy skin tissue and keratinocytes were isolated from anonymised human abdominal tissue discarded during routine surgery at Queen Victoria Hospital, UK (NHS Trust) with consent from patients and full ethical approval from the National Regional Ethics Service (REC:06/Q1907/81). While no exclusion criteria were used, all donors (*n* = 6) were female aged 44–66 years (average age of 53). To isolate keratinocytes, full-thickness dermatomed skin slices were first incubated (37 °C, 5% CO_2_) in dispase II (2U/ml) for 30 min, the epidermis isolated and incubated (10 min) in 0.5% trypsin to free keratinocytes. Isolated keratinocytes were collected by centrifugation (500G, 5 min) in 10 ml DMEM (10% FCS, 1% penicillin/streptomycin) and cultured in CnT-O7S KC defined medium (CalTag, UK) supplemented with 1% penicillin/streptomycin at a seeding density of 2 × 10^6^ per T75 flask. The culture medium was replaced every three days and cells were used at 85% confluence and between passages 1 and 3. All enzymes used were prepared in Hank's Balanced Salt Solution (HBSS) supplemented with 10% penicillin/streptomycin (Gibco Life Technologies, UK).

### Keratinocyte seeding and culture on skin explants

**Fig. 1:**
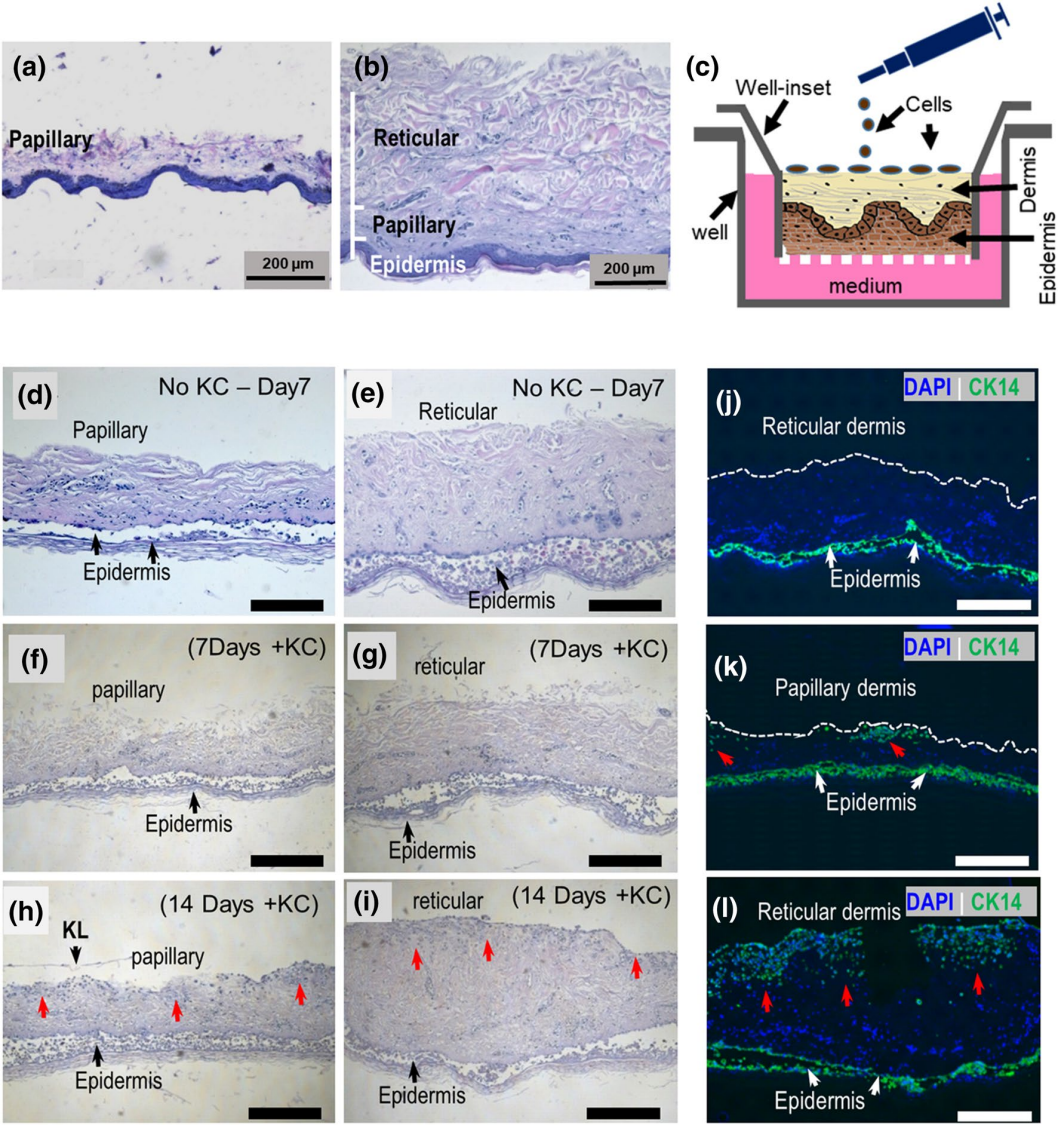
Skin explants cultured upside down at an air–liquid interface for 14 day support keratinocyte proliferation and tissue infiltrations. Representative H&E stains of split-thickness skin shaved to **a** papillary and **b** the reticular dermal layer depth. **c** Schematic representation of the upside-down skin explant culture and keratinocyte seeding onto exposed dermis. **d** HE stained skin explants after 7 and 14 days in culture. BM is membrane and KL is keratinised layer. **e** Representative fluorescence micrographs of anti-CK14 stained explant sections after 14 days in culture with and without keratinocytes (red arrows). Epidermal keratinocytes (bottom green fluorescence) were not evaluated. Scale bars are 200 µm.

Punch biopsies (1 cm diameter) of split-thickness skin shaved to a depth of 300 (for papillary dermis explant) and 600 µm (for reticular dermis explant) (Fig. 1a, b) were placed, with epidermis still attached, in ThinSert^®^ membraned cell culture inserts (Greiner Bio-One Ltd., Stonehouse, UK) upside down (dermal-side up) and cultured in 6-well plates for 7–21 days. Autologous keratinocytes were then seeded (1 × 10^3^ cell per biopsy) on the exposed dermal side (Fig. 1c) and the cultures supplemented with 2 ml culture media allowing for an air–liquid interface to be created at the surface. Three technical replicates were performed. Culture conditions were 37 °C and 5% CO_2_ with culture medium changes every 2 days.

### Histological stains

After 0, 3, 7, 14, and 21 days in culture, skin explants were fixed with 4% neutral-buffered formaldehyde for 10 min, processed in paraffin, and wax embedded. Tissue sections (8 µm thick) were stained with haematoxylin and eosin (H&E) for morphological evaluation and with Movat-Russel’s Modified Pentachrome [3] to resolve different ECM features. Between stains, slides were rinsed in alcoholic and acidic solutions.

### Immunohistochemistry

Immunostaining for cytokeratin 14 (CK-14), collagen VII (Col VII), laminin, and connexin 26 and 43 (cx26/43) was performed with antibodies specific for each protein (list below). For antigen retrieval, sections were incubated (20 min, 37 °C) with proteinase K (Abcam, Cambridge, UK) and permeabilized with 0.1% Triton X100 (Sigma-Aldrich, St. Louis, USA) in PBS with Tween 20 and 1% normal goat serum (PBST) (30 min, 30 °C). Non-specific binding was then blocked with 10% normal goat serum in PBST (Vector Laboratories Inc., Burlingame, USA) (all samples) and 1% Bovine Serum Albumin (BSA) in PBS (col VII section) (2 h, 20 °C). Sections were then incubated with primary antibodies for either 2 h, ≈ 20 °C (laminin and, cx 26 and anti 43) and overnight (≈ 12 h) (CK-14 and col vii), followed by secondary antibodies for 30 min (anti-rabbit) and 2 h (anti-mouse) at room temperature.

Primary mouse monoclonal antibodies used: anti-CK14 (LL002, dilution 1:1000, Abcam, Cambridge, UK), anti-col vii (LH7.2, dilution 1:1000, in PBST, Abcam, Cambridge, UK), and anti-Cx26 (CX-1E8, dilution 1:2000 in PBS, Invitrogen, Carlsbad, USA). Primary rabbit polyclonal antibodies used: anti-laminin (AB30320, dilution 1:200 in PBST, Abcam, Cambridge, UK) and anti-Cx43 (SAB4501173**,** dilution of 1:2000 in PBST, Sigma-Aldrich, St. Louis, USA). The secondary antibodies used: monoclonal DyLight^®^-488 (Green) anti-mouse IgG and polyclonal DyLight^®^-549 (Red) anti-rabbit Ig, both at a dilution of 1:200 in PBS (Vector Laboratories Inc., Burlingame, USA).

All sections were re-mounted in Vectashield + DAPI antifade mounting medium (Vector Laboratories Inc., Burlingame, USA) and imaged on a Zeiss Axioscope A.1 microscope equipped with AxioVision v4.8.2 software (Carl Zeiss AG, Oberkochen, Germany). Positive immunostaining for laminin and the number of cx 26 and 43 positive cells per mm^2^ of tissue within a depth of 200 µm from the exposed surface in two sections of each sample were quantified using ImageJ 1.52C software. Cells and positive stained features in the epidermis and basement membrane within 100 µm distance from the basement membrane into the dermis were excluded from the analysis. All laminin positive stain in blood vessels was excluded wherever it was detected in tissue.

## Results and discussion

Seeded keratinocytes infiltrated the reticular and papillary layers differently (Fig. 1). Since enzymatic removal of the epidermis from skin explants can potentially adulterate the ECM and cells, skin explants were cultured with the epidermis still attached (Fig. 1c). However, in line with findings by others [9, 13], the epidermis showed spongiosis-like features with marked detachment of the spinous layer in all explants after 7 days in culture (Fig. 1d–i). No differences across all samples were observed after 3 days in culture (data not shown). This was also observed in explants cultured epidermal-side up (data not shown). However, the dermal layers remained intact for 14 days with loosening of the exposed surface after 7 days (Fig. 1f, g) and poor reproducibility beyond 21 days partly due to coiling up of the explant, which enclosed the exposed surface (data not shown). However, human dermal explants cultured at a liquid–air interface in vitro have been shown by others to remain viable after 7 days [12] and in some cases 75 days [11]. While no keratinocytes were detected on explant surfaces after 7 days (Fig. 1j), increased keratinocyte infiltration in both papillary and reticular samples was eventually observed after 14 days, suggesting proliferation (Fig. 1h, i). However, while seeded keratinocytes appeared to form clusters in the papillary layer with shallower tissue infiltration (Fig. 1k), their spread was homogeneous and deeper in the reticular dermis with a characteristic intact single-cell-layer coverage of the surface (Fig. 1i, l). This varied keratinocyte distribution may be due to differences in the physical features of the two dermal layers, though others have demonstrated variation in ECM biochemistry, including that which affects keratinocyte proliferation and migration (i.e., hyaluronan and other proteins) as well as ECM remodelling, across dermal layers [11].

**Fig. 2:**
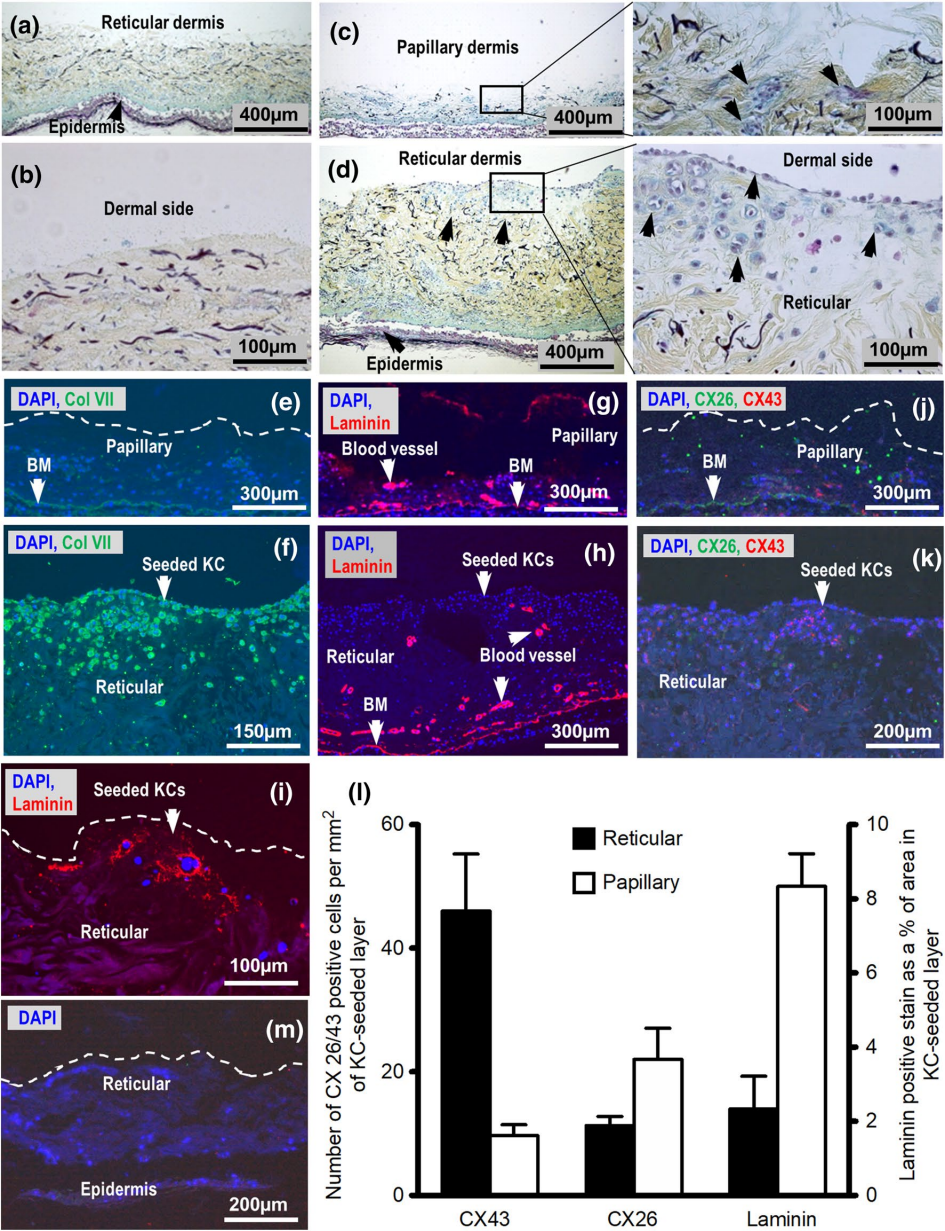
Keratinocytes cultured on the papillary and reticular dermal surfaces at the liquid–air interface for 14 days remodelled dermal-ECM differently. Movat-Russell’s modified pentachrome stain showing: **a** Control sections cultured with no keratinocytes seeded and **b** magnification of the control surface; Papillary (**c**) and reticular (**d**) dermal sections seeded with keratinocytes. Representative micrographs of the papillary and reticular sections stained with col VII (**e**, **f** respectively), laminin (**g**, **h** respectively), and both Cx26 and 43 (**j**, **k** respectively). (i) Representative laminin stained reticular section after 21 days in culture. (l) Graphical representation of Cx26/43 and laminin immunostains of the KC seeded layer. Laminin in blood vessels and basement membrane (BM) was not factored in (*n* = 3 and error bars = SEM). (M) Representative control section with no antibody.

Indeed, after seeding of keratinocytes, the ECM features in the reticular and papillary dermal layers showed notable differences (Fig. 2). While the normal structure and distribution of ECM features such as elastin, collagen, reticular fibres, ground substance, and GAGs remained unchanged in control samples after 14 days in culture (Fig. 2a, b), newly deposited ground substance and or GAGs (light blue/green stain) were observed around seeded keratinocytes in both the papillary (Fig. 2c) and reticular samples (Fig. 2d). The localisation of the new ground substance and GAG positive stain, typically found in the papillary layer, as well as col VII, a basement membrane component (Fig. 2e, f), around seeded keratinocytes within the reticular dermal layer suggest the involvement of seeded keratinocytes in ECM remodelling. Indeed, the presence of keratinocytes has been shown to lead to the production of keratinocyte growth factors and GAGs (i.e., hyaluronan) by fibroblasts that promote keratinocyte migration [2, 4, 7], ECM production, and re-epithelialisation in wound healing through an interplay of complex autocrine and paracrine pathways. It has been shown by others that keratinocyte-derived IL-1α and β control fibroblast expression of keratinocyte growth factor (KGF/KGF7), which, in turn, enhances keratinocyte expression of tumour necrosis factor-*α* (TNF-*α*) resulting in collagen synthesis by fibroblasts as well as expression of MMPs and TIMPs, and synthesis of ECM components by both keratinocytes and fibroblasts [2, 7]. Moreover, in the immunostain for laminin (Fig. 2g–i) and intracellular gap junction connections Cx26 and Cx43 (Fig. 2j, k), more new laminin and Cx26-positive stains were detected in the papillary than in the reticular tissue sections around seeded keratinocytes. However, significantly more Cx43-positive stain (*p* < 0.05) was detected in the reticular tissue sections than in the papillary (Fig. 2 l). Cx26 and Cx43 are known to directly affect the proliferation and migration of keratinocytes and fibroblast [7, 15] and the observed differential expression of these connexins suggests differences in cellular activities.

Since there was no evidence of native keratinocyte migrating from epidermis to the exposed dermal surface in this work, the positive stain for CK14 along with newly deposited col VII and GAGs within and on the exposed dermal layers around keratinocytes suggests that the skin explants supported the production of robust layer-seeded keratinocytes. However, the differences in the nature of spread of keratinocytes, ground substance, and basement membrane-associated proteins (col VII and laminin) suggest differences in physico-chemical interaction between the seeded keratinocytes and features of the dermal layers. Furthermore, the difference in Cx26 and Cx43 expression between the dermal layers suggests possible difference in intercellular interaction and possible fibroblast involvement. Indeed, the role of these connexins in the regulation of fibroblast and keratinocyte proliferation, migration, and activity in both normal and diseased skin are well established [2].

## Conclusions

Although the cellular and ECM differences in skin layers are well established, their clinical impact in wound interventions remains unclear. These findings in ex vivo skin explant cultures are in line with suggestions that the varying dermal layer features (ECM and potentially fibroblast) may play key roles in re-epithelialisation and should perhaps be considered in the design and development of biomaterials for wound applications as well as in wound healing interventions such as autologous keratinocyte grafting [15]. Indeed, the differential expression of these ECM features in the two dermal layers around newly seeded keratinocytes suggests potential imperative features and mechanisms in wound healing interventions. However, detailed and specific analysis of the cellular pathways, proliferation, and activities involved are necessary for conclusive clinical-efficacy characterisation.
